# The potential risks of C-C chemokine receptor 5-edited babies in bone development

**DOI:** 10.1038/s41413-019-0044-0

**Published:** 2019-01-29

**Authors:** Yong Xie, Shaohua Zhan, Wei Ge, Peifu Tang

**Affiliations:** 1 Department of Orthopedics, Chinese PLA General Hospital, Beijing, China; 2 State Key Laboratory of Medical Molecular Biology and Department of Immunology, Institute of Basic Medical Sciences, Chinese Academy of Medical Sciences, Beijing, China

**Keywords:** Bone, Physiology

## Introduction

Hutter et al.^1^ first reported that a bone marrow transplant using stem cells derived from a donor with homozygous *CCR5* delta32 gene mutation remained HIV-positive but virus-free (below the limits of detection) after halting antiretroviral therapy. Since this observation in 2009, mutation of the *CCR5* gene has become an important target in the prevention and treatment of HIV infection. The CRISPR-Cas9 system, which has been called the biggest biotech discovery in the history of molecular biology, can be used for precise genome engineering with the aim of treating genetic disorders. Currently, the application of gene-editing tools, such as CRISPR-Cas9, for genetic engineering of embryos for use in assisted reproduction is prohibited in much of Europe, the United States, and China.^2,3^ However, at the Second International Summit on Human Genome Editing in Hong Kong (http://www.nationalacademies.org/), Jiankui He claimed that his team had used CRISPR-Cas9 systems to successfully edit the *CCR5* gene in twin baby girls, Lulu and Nana. In Lulu, one copy of exon 3 in the *CCR5* gene has an inserted base, with the other copy missing four bases. In Nana, a 15-nucleotide deletion (delta15) within one copy of *CCR5* was described, with the other copy of the *CCR5* gene remaining intact.

The *CCR5* gene is located at chromosome region 3p21.31^4^ and comprises three exons, two introns and two promoters.^5^ The C-C chemokine receptor 5 (CCR5) protein encoded by the *CCR5* gene consists of 352 amino acids^6^ and is composed of a conserved, N-terminal seven trans-membrane domain and a C-terminal tail.^7^ This structure is important for the binding of HIV glycoprotein receptors to host cells and HIV co-receptor CD4 activity.^8^ Samson et al. found that the second extracellular loop of CCR5 is specifically affected by delta32 mutations in exon 3, which result in the absence of the final three trans-membrane domains in addition to regions involved in G-protein interaction and signal transduction. In CD4+ cells, this mutation inhibits CCR5 protein expression on the cell surface, thereby preventing HIV envelope fusion.^9^ Moreover, the presence of the mutant delta32 protein in the endoplasmic reticulum inhibits transport of the wild-type CCR5 protein to the cell surface via a trans-dominant mechanism.^10^ Because most strains of HIV use CCR5 to enter host cells, the deletion of both copies of the *CCR5* gene (not one copy) protects against HIV infection.^11,12^ Thus, Nana would still be susceptible to HIV infection. Although He demonstrated that Lulu was homozygous for the disrupted *CCR5* gene, this child may also be genetically mosaic, which means that Lulu may carry some edited cells and some unedited cells. Furthermore, although He claimed an absence of dangerous off-target mutations in both twins based on single cell sequencing studies, these results were not peer-reviewed and confirmed by an independent team. Therefore, Lulu’s genetic status should be continually monitored throughout her life, and it is possible that she may encounter unpredictable disorders in the future.

### Role of *CCR5* deficiency in diseases

Individuals who are naturally homozygous for the delta32 mutation, which abolishes CCR5 expression, are generally healthy and at no apparent disadvantage.^8^ However, apart from the protective effects against HIV infection, the impacts of this mutation, positive or negative, on other diseases are open to debate.^13^ To date, several studies have indicated that *CCR5* delta32 mutations provide significant resistance to smallpox,^14^ in addition to enhancing certain forms of memory^15^ but also render individuals more vulnerable to influenza^16^ and the West Nile virus.^17^ In mice, *CCR5* deficiency exacerbates stroke-related brain injury.^18^ CCR5 is thought to mediate pro-inflammatory effects in the pathogenesis of rheumatoid arthritis (RA).^19^ However, Fleishaker et al.^20^ reported that a CCR5 antagonist (maraviroc), which has been approved for use in HIV patients, was ineffective in treating patients with RA who had not responded to methotrexate (MTX). Moreover, a double-blind, placebo-controlled trial in 2015 found that maraviroc was associated with reduced bone loss at the hip and lumbar spine of HIV-infected patients.^21^ Other studies demonstrated direct roles of CCR5 in osteoclastogenesis and osteoclast-osteoblast communication.^22,23^ These clinical and basic investigations highlight the skeletal effects associated with the functional loss of CCR5.^24^

### CCR5 deficiency in osteoclast differentiation and function

Previous epidemiological studies have suggested that disrupted CCR5 is associated not only with a lower frequency of HIV transmission but also with a reduced incidence and severity of bone-destructive diseases.^25,26^ These studies demonstrate that CCR5 is a pivotal factor in bone development and regulation.^21^ Compared with wild-type alveolar bone, *CCR*-deficiency was shown to be associated with an increased number of tartrate-resistant acid phosphatase (TRAP)-positive osteoclasts with upregulated osteoclastic markers in a model of orthodontic tooth movement.^27^ In 2017, antibody-mediated CCR5 blockade was shown to have a detrimental effect on human osteoclast function. Moreover, *CCR5*-deficient mice were found to be resistant to bone loss induced by receptor activator of nuclear factor kappa-B ligand (RANKL) via a mechanism that is independent of inflammatory and immunomodulatory pathways.^28^ These *CCR5*-deficient mice also presented increased numbers of osteoclast precursors and osteoclasts exhibiting disorganized cellular adhesion and a reduced bone resorptive ability, which was also accompanied by downregulated RANKL-induced phosphorylation of the proto-oncogene tyrosine-protein kinase Src (SRC) and protein-tyrosine kinase 2-β (PTK2B). Such integrin-mediated signaling complexes regulate the actin cytoskeleton reorganization required for cell polarization and adhesion by activation of Rho family GTPases, such as transforming protein RhoA (RHOA) and ras-related protein Rac1 (RAC1).^28^ These findings demonstrate that CCR5 is required for the focal adhesion-mediated signaling involved in cellular locomotion, podosome-related sealing zone organization, and bone resorptive activity, thereby elucidating the essential role of CCR5 in bone-destructive conditions through the functional regulation of mature osteoclasts. In contrast, the latest reports in 2018 demonstrated that CCR5 expression was rapidly reduced by RANKL treatment during osteoclastogenesis but was recovered by the administration of IFN-γ. RANKL-induced CCR5 downregulation is mediated by mitogen-activated protein kinase (MAPK) in osteoclast precursors and promotes osteoclastogenesis.^29^ The master transcription factor of the MAPK pathway, nuclear factor of activated T-cells, cytoplasmic 1 (NFATc1), possibly regulates the transcription of CCR5 by binding to the *CCR5* promoter.^30^ However, CCR5 blockade may not completely impact osteoclast differentiation due to other chemokine receptors that are similarly upregulated by IFN-γ^31^ and downregulated by RANKL treatment.^32^

### *CCR5* deficiency in immune cells and bone regulation

**Fig. 1:**
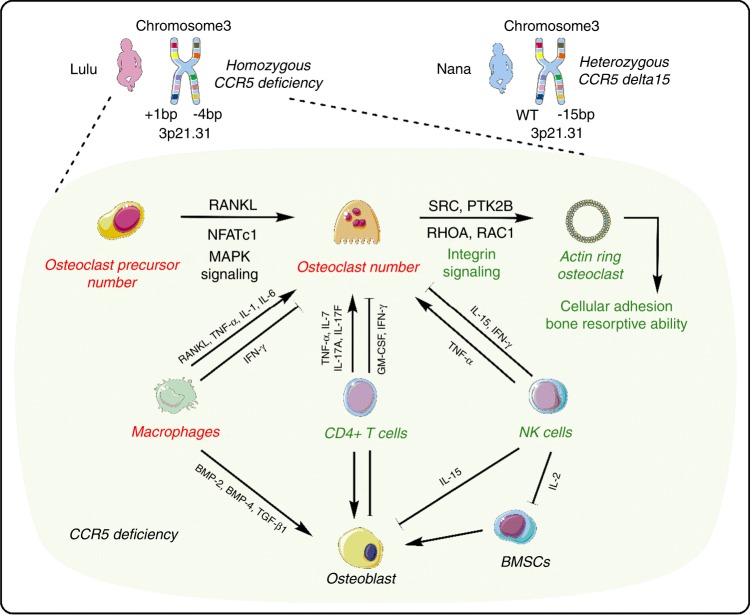
The potential molecular mechanisms for vulnerability of a homozygous *CCR5-*deficient individual to bone disorders. The *CCR5* gene is located at chromosome region 3p21.31. In Lulu, one copy of exon 3 in the *CCR5* gene has an inserted base, with the other copy missing four bases. In Nana, a 15-nucleotide deletion (delta15) within one copy of *CCR5* was described, with the other copy of the *CCR5* gene remaining intact. Thus, Lulu may be a homozygous *CCR5*-deficient baby. Although the number of osteoclast precursors and osteoclasts were upregulated in *CCR5*-deficiency models, their cellular adhesion and bone resorptive ability were downregulated. Red characters indicate upregulated cell number or biological function, whereas green characters indicate downregulated cell number or biological function. Solid black arrows represent the promotion of cellular processes, and solid T bars represent the inhibition of cellular processes. CCR5: C-C chemokine receptor type 5; RANKL: Receptor activator of nuclear factor kappa-Β ligand; NFATc1: Nuclear factor of activated T-cells, cytoplasmic 1; PTK2B: Protein-tyrosine kinase 2-β; RAC1: Ras-related protein Rac1; RHOA: Transforming protein RhoA; MAPK: Mitogen-activated protein kinase; GM-CSF: Granulocyte-macrophage colony-stimulating factor; BMP-2: Bone morphogenetic protein 2; BMP-4: Bone morphogenetic protein 4; BMSCs: Bone marrow stromal cells. Original elements used in this diagram are from Servier Medical Art (http://smart.servier.com/)

CCR5 is expressed on various immune cells including T-cells, macrophages and natural killer (NK) cells.^33^ Numerus studies have demonstrated high levels of integration of the skeletal and immune systems.^34^ In addition to its role in chemotaxis, CCR5 signaling has been implicated in T cell differentiation and enhances adaptive immune responses. For instance, CCR5 enhances T lymphocyte co-stimulation and CD4 + T cell cytokine release. Activated T-cells produce RANKL and induce bone loss.^35^ CD4 + T-cells inhibit osteoclastogenesis by expressing GM-CSF and IFN-γ^36^. In contrast, under IL-15 stimulation, CD4 + T-cells also express TNF-α to promote osteoclastogenesis.^37^ IL-7 produced by T-cells also promotes osteoclast formation by upregulating T cell-derived cytokines, such as TNF-α^38^. T helper 17 (Th17) cells stimulate osteoblast differentiation through the secretion of their main pro-inflammatory cytokines, IL-17A and IL-17F.^39^ Therefore, *CCR5* deficiency in T-cells might reduce the secretion of cytokines involved in the regulation of osteoclast and osteoblast differentiation. In a mouse renal allograft model, *CCR5* deficiency resulted in accumulation of alternatively activated macrophages.^40^ This activation of macrophages in the absence of CCR5 was demonstrated by normal expression of inducible nitric oxide synthase (iNOS) and production of the cytokine IFN-γ^40^. Macrophages could regulate RANKL-induced osteoclastogenesis by stimulation of pro-inflammatory cytokines such as TNF-α, IL-1, and IL-6^41-45^. However, IFN-γ inhibits osteoclastogenesis through the subsequent rapid degradation of TRAF6.^46^ Thus, macrophages promote or suppress osteoclast activity through the secretion of TNF-α, IL-1, IL-6, and IFN-γ. Macrophages also support osteoblast differentiation and proliferation through the release of cytokines including BMP-2, BMP-4, and TGF-β1.^47,48^ Notably, CCR5-deficient mice exhibited a significant decrease in the number of CD3 + NK cells. With a normal apoptosis rate, the potential proliferation of NK cells derived from *CCR5*-deficient mice was reduced.^49^ Moreover, those NK cells showed significantly reduced adherence to target cells including osteoclasts or osteoblasts, in addition to lower killing efficiency.^50^ Other results indicated that the development and trafficking of NK cells are regulated by prolonged inhibition of CCR5 signaling.^49^ Bone marrow stromal cells (BMSCs) are bound and killed by IL-2 activated NK cells.^51^ Meanwhile, the NK cells release IFN-γ and TNF-α, which regulate osteoclast differentiation. Furthermore, NK cells also promote apoptosis of osteoblasts and osteoclasts through IL-15 stimulation.^52,53^ A reduced number and function of NK cells could affect the life span of osteoclasts and osteoblasts (Fig. 1).

## Discussion and concerns

Bone growth and development are involved in bone modeling, which occurs predominantly in the prepubertal period, and bone remodeling, which occurs throughout life after sexual maturity. Bone modeling defines skeletal growth and development and, in this context, is responsible for reshaping the bone during growth, with bone formation predominating over resorption. This process is essential for bone health and requires osteoclasts and osteoblasts to function independently in distinct sites.^54^ In contrast, during bone remodeling, osteoclasts and osteoblasts work sequentially in the same location.^55^ This process is characterized by activation, resorption, reversal and formation phases. In the activation phase, osteoclasts are recruited, whereas osteoclasts resorb bone in the resorption phase. In the reversal phase, osteoclasts undergo apoptosis and osteoblasts are recruited, and in the formation phase, osteoblasts generate new organic bone matrix, which is subsequently mineralized.^56^ Osteoclasts, which are derived from hematopoietic stem cells (HSCs), are unique in their ability to resorb bone matrices and are currently thought to have precursors in common with macrophages.^57^ Other than its effects on immune cells, *CCR5* deficiency primarily influences osteoclast function; thus, being homozygous for the disrupted *CCR5* gene, Lulu may be affected by risks to her bone development.

The specific architecture of the cytoskeleton of osteoclasts allows polarization and adhesion of their unique resorptive machinery to the bone surface, where an isolated resorptive microenvironment is sealed by an actin ring and integrin-based podosomes, known as the sealing zone.^58^ The sealing zone is a highly dynamic structure, undergoing cycles of assembly and disassembly.^58^ Loss of CCR5 function causes abrogated actin ring formation^59^ of mature osteoclasts due to the rearrangement of podosomes, which is also accompanied by the dissociation of focal adhesions.^28^ Integrins are heterodimeric cell surface receptors that mediate cell–cell and cell–matrix interactions.^60^*CCR5* deficiency seems to interfere with the organization and function of integrin-associated adhesion and migration of both osteoclasts and immune cells. The coordinated regulation of these cells is critical for maintaining physiological bone modeling and remodeling, ensuring proper bone development and health.

The dispensable physiological role of CCR5 is highlighted by the apparent health and lack of abnormalities in delta32-homozygous individuals. However, it can be speculated that these individuals have adapted to this deficiency by the evolution of alternative receptors or structures required for immune and other functions since the ancestral acquisition of this mutation thousands of years ago. Thus, artificially inducing a null CCR5 phenotype in the baby Lulu may have unforeseen consequences. The potential risks to bone development in CCR5-edited babies are hard to predict, and only time will reveal the long-term effects of *CCR5* deficiency on the affected individual.

## References

[1] Hutter G (2009). Long-term control of HIV by CCR5 Delta32/Delta32 stem-cell transplantation. New Engl. J. Med..

[2] Wang, C. et al. Gene-edited babies: Chinese Academy of Medical Sciences’ response and action. *Lancet*. 10.1016/S0140-6736(18)33080-0 (2018). 10.1016/S0140-6736(18)33080-0 30522918

[3] National Academies of Sciences. *Human Genome Editing: Science, Ethics, and Governance* (National Academies Press, Washington, DC, 2017). 28796468

[4] Haworth, K. G., Peterson, C. W. & Kiem, H. P. CCR5-edited gene therapies for HIV cure: Closing the door to viral entry. *Cytotherapy* **19**, 1325–1338 (2017). 10.1016/j.jcyt.2017.05.013 28751153

[5] Martin-Blondel G, Brassat D, Bauer J, Lassmann H, Liblau RS (2016). CCR5 blockade for neuroinflammatory diseases--beyond control of HIV. Nat. Rev. Neurol..

[6] Brelot, A. & Chakrabarti, L. A. CCR5 revisited: how mechanisms of HIV entry govern AIDS pPathogenesis. *J. Mol. Biol.* **430**, 2557–2589 (2018). 10.1016/j.jmb.2018.06.027 29932942

[7] Vangelista, L. & Vento, S. The expanding therapeutic perspective of CCR5 blockade. *Front. Immunol.* **8**, 1981(2017). 10.3389/fimmu.2017.01981 PMC5770570 29375583

[8] Barmania, F. & Pepper, M. S. C-C chemokine receptor type five (CCR5): An emerging target for the control of HIVinfection. *Appl. Transl. Genom.* **2**, 3–16 (2013). 10.1016/j.atg.2013.05.004 PMC5133339 27942440

[9] Samson M (1996). Resistance to HIV-1 infection in caucasian individuals bearing mutant alleles of the CCR-5 chemokine receptor gene. Nature.

[10] Agrawal L (2004). Role for CCR5Delta32 protein in resistance to R5, R5X4, and X4 human immunodeficiency virus type 1 in primary CD4 + cells. J. Virol..

[11] Guignard F, Combadiere C, Tiffany HL, Murphy PM (1998). Gene organization and promoter function for CC chemokine receptor 5 (CCR5). J. Immunol..

[12] Liu R (1996). Homozygous defect in HIV-1 coreceptor accounts for resistance of some multiply-exposed individuals to HIV-1 infection. Cell.

[13] Lopalco L (2010). CCR5: from natural resistance to a new anti-HIV strategy. Viruses.

[14] Galvani AP, Slatkin M (2003). Evaluating plague and smallpox as historical selective pressures for the CCR5-Delta 32 HIV-resistance allele. Proc. Natl Acad. Sci. USA.

[15] Zhou, M. et al. CCR5 is a suppressor for cortical plasticity and hippocampal learning and memory. *eLife* **5**, 10.7554/eLife.20985 (2016). 10.7554/eLife.20985 PMC5213777 27996938

[16] Falcon A (2015). CCR5 deficiency predisposes to fatal outcome in influenza virus infection. J. Gen. Virol..

[17] Glass WG (2006). CCR5 deficiency increases risk of symptomatic West Nile virus infection. J. Exp. Med..

[18] Sorce, S. et al. Increased brain damage after ischaemic stroke in mice lacking the chemokine receptor CCR5. *Br. J. Pharmacol.* **160**, 311–321 (2010). 10.1111/j.1476-5381.2010.00697.x PMC2874853 20423342

[19] Takeuchi T, Kameda H (2012). What is the future of CCR5 antagonists in rheumatoid arthritis?. Arthritis Res. Ther..

[20] Fleishaker DL (2012). Maraviroc, a chemokine receptor-5 antagonist, fails to demonstrate efficacy in the treatment of patients with rheumatoid arthritis in a randomized, double-blind placebo-controlled trial. Arthritis Res. Ther..

[21] Taiwo BO (2015). Less bone loss with maraviroc- versus tenofovir-containing antiretroviral therapy in the AIDS Clinical Trials Group A5303 Study. Clin. Infect. Dis..

[22] Han JH (2001). Macrophage inflammatory protein-1alpha is an osteoclastogenic factor in myeloma that is independent of receptor activator of nuclear factor kappaB ligand. Blood.

[23] Yano, S. et al. Functional expression of beta-chemokine receptors in osteoblasts: role of regulated upon activation, normal T cell expressed and secreted (RANTES) in osteoblasts and regulation of its secretion by osteoblasts and osteoclasts. *Endocrinology* **146**, 2324–2335 (2005). 10.1210/en.2005-0065 15718270

[24] Oba Y (2005). MIP-1alpha utilizes both CCR1 and CCR5 to induce osteoclast formation and increase adhesion of myeloma cells to marrow stromal cells. Exp. Hematol..

[25] Pokorny V (2005). Evidence for negative association of the chemokine receptor CCR5d32 polymorphism with rheumatoid arthritis. Ann. Rheum. Dis..

[26] Prahalad S (2006). Association of two functional polymorphisms in the CCR5 gene with juvenile rheumatoid arthritis. Genes Immun..

[27] Andrade I (2009). CCR5 down-regulates osteoclast function in orthodontic tooth movement. J. Dent. Res..

[28] Lee JW (2017). The HIV co-receptor CCR5 regulates osteoclast function. Nat. Commun..

[29] Lee D (2018). CCL4 enhances preosteoclast migration and its receptor CCR5 downregulation by RANKL promotes osteoclastogenesis. Cell Death Dis..

[30] Wierda RJ, van den Elsen PJ (2012). Genetic and epigenetic regulation of CCR5 transcription. Biology.

[31] Zella D (1998). Interferon-gamma increases expression of chemokine receptors CCR1, CCR3, and CCR5, but not CXCR4 in monocytoid U937 cells. Blood.

[32] Lean JM, Murphy C, Fuller K, Chambers TJ (2002). CCL9/MIP-1gamma and its receptor CCR1 are the major chemokine ligand/receptor species expressed by osteoclasts. J. Cell. Biochem..

[33] Rucker J (1996). Regions in beta-chemokine receptors CCR5 and CCR2b that determine HIV-1 cofactor specificity. Cell.

[34] Okamoto K (2017). Osteoimmunology: the conceptual framework unifying the immune and skeletal systems. Physiol. Rev..

[35] Kong YY (1999). Activated T cells regulate bone loss and joint destruction in adjuvant arthritis through osteoprotegerin ligand. Nature.

[36] Shinoda K (2003). Resting T cells negatively regulate osteoclast generation from peripheral blood monocytes. Bone.

[37] McInnes IB, Leung BP, Sturrock RD, Field M, Liew FY (1997). Interleukin-15 mediates T cell-dependent regulation of tumor necrosis factor-α production in rheumatoid arthritis. Nat. Med..

[38] Roato I (2006). IL-7 up-regulates TNF-alpha-dependent osteoclastogenesis in patients affected by solid tumor. PLoS ONE.

[39] Croes, M. et al. Proinflammatory T cells and IL-17 stimulate osteoblast differentiation. *Bone* **84**, 262–270 (2016). 10.1016/j.bone.2016.01.010 26780388

[40] Dehmel S (2010). Chemokine receptor Ccr5 deficiency induces alternative macrophage activation and improves long-term renal allograft outcome. Eur. J. Immunol..

[41] Azuma Y, Kaji K, Katogi R, Takeshita S, Kudo A (2000). Tumor necrosis factor-α induces differentiation of and bone resorption by osteoclasts. J. Biol. Chem..

[42] Udagawa, N et al. Interleukin (IL)-6 induction of osteoclast differentiation depends on IL-6 receptors expressed on osteoblastic cells but not on osteoclast progenitors. *J. Exp. Med.* **182**, 1461–1468 (1995). 10.1084/jem.182.5.1461 PMC2192181 7595216

[43] Yoshitake F, Itoh S, Narita H, Ishihara K, Ebisu S (2008). Interleukin-6 directly inhibits osteoclast differentiation by suppressing receptor activator of NF-kappaB signaling pathways. J. Biol. Chem..

[44] Lee YM, Fujikado N, Manaka H, Yasuda H, Iwakura Y (2010). IL-1 plays an important role in the bone metabolism under physiological conditions. Int. Immunol..

[45] Takayanagi H (2007). Osteoimmunology: shared mechanisms and crosstalk between the immune and bone systems. Nat. Rev. Immunol..

[46] Takayanagi H (2000). T-cell-mediated regulation of osteoclastogenesis by signalling cross-talk between RANKL and IFN-gamma. Nature.

[47] Champagne CM, Takebe J, Offenbacher S, Cooper LF (2002). Macrophage cell lines produce osteoinductive signals that include bone morphogenetic protein-2. Bone.

[48] Fromigue O, Marie PJ, Lomri A (1998). Bone morphogenetic protein-2 and transforming growth factor-beta2 interact to modulate human bone marrow stromal cell proliferation and differentiation. J. Cell. Biochem..

[49] Weiss, I. D. et al. Ccr5 deficiency regulates the proliferation and trafficking of natural killer cells under physiological conditions. *Cytokine* **54**, 249–257 (2011). 10.1016/j.cyto.2011.01.011 21376626

[50] Ajuebor, M. N. et al. CCR5 deficiency drives enhanced natural killer cell trafficking to and activation within the liver in murine T cell-mediated hepatitis. *Am. J. Pathol.* **170**, 1975–1988 (2007). 10.2353/ajpath.2007.060690 PMC1899451 17525265

[51] Poggi A (2005). Interaction between human NK cells and bone marrow stromal cells induces NK cell triggering: role of NKp30 and NKG2D Receptors. J. Immunol..

[52] Takeda H (2014). Effect of IL-15 and natural killer cells on osteoclasts and osteoblasts in a mouse coculture. Inflammation.

[53] Feng S (2015). Interleukin-15-activated natural killer cells kill autologous osteoclasts via LFA-1, DNAM-1 and TRAIL, and inhibit osteoclast-mediated bone erosion in vitro. Immunology.

[54] Langdahl B, Ferrari S, Dempster DW (2016). Bone modeling and remodeling: potential as therapeutic targets for the treatment of osteoporosis. Ther. Adv. Musculoskelet. Dis..

[55] Henriksen K, Karsdal MA, Martin TJ (2014). Osteoclast-derived coupling factors in bone remodeling. Calcif. Tissue Int..

[56] Teti A (2011). Bone development: overview of bone cells and signaling. Curr. Osteoporos. Rep..

[57] Kikuta J, Ishii M (2013). Osteoclast migration, differentiation and function: novel therapeutic targets for rheumatic diseases. Rheumatology.

[58] Batsir S, Geiger B, Kam Z (2017). Dynamics of the sealing zone in cultured osteoclasts. Cytoskeleton.

[59] Georgess D, Machuca-Gayet I, Blangy A, Jurdic P (2014). Podosome organization drives osteoclast-mediated bone resorption. Cell Adhes. Migr..

[60] Lin, T. H. et al. Inhibition of osteoporosis by the alphavbeta3 integrin antagonist of rhodostomin variants. *Eur. J. Pharmacol.* **804**, 94–1011 (2017). 10.1016/j.ejphar.2017.03.019 28315346

